# The Importance of the Derivative in Sex-Hormone Cycles: A Reason Why Behavioural Measures in Sex-Hormone Studies Are So Mercurial

**DOI:** 10.1371/journal.pone.0111891

**Published:** 2014-11-26

**Authors:** Adam McNamara, Kaylee Moakes, Philip Aston, Christine Gavin, Annette Sterr

**Affiliations:** 1 School of Psychology, University of Surrey, Guildford, United Kingdom; 2 Department of Mathematics, University of Surrey, Guildford, United Kingdom; Sun Yat-sen University, China

## Abstract

To study the dynamic changes in cognition across the human menstrual cycle, twenty, healthy, naturally-cycling women undertook a lateralized spatial figural comparison task on twelve occasions at approximately 3–4 day intervals. Each session was conducted in laboratory conditions with response times, accuracy rates, eye movements, salivary estrogen and progesterone concentrations and Profile of Mood states questionnaire data collected on each occasion. The first two sessions of twelve for the response variables were discarded to avoid early effects of learning thereby providing 10 sessions spread across each participant's complete menstrual cycle. Salivary progesterone data for each participant was utilized to normalize each participant's data to a standard 28 day cycle. Data was analysed categorically by comparing peak progesterone (luteal phase) to low progesterone (follicular phase) to emulate two-session repeated measures typical studies. Neither a significant difference in reaction times or accuracy rates was found. Moreover no significant effect of lateral presentation was observed upon reaction times or accuracy rates although inter and intra individual variance was sizeable. We demonstrate that hormone concentrations alone cannot be used to predict the response times or accuracy rates. In contrast, we constructed a standard linear model using salivary estrogen, salivary progesterone and their respective derivative values and found these inputs to be very accurate for predicting variance observed in the reaction times for all stimuli and accuracy rates for right visual field stimuli but not left visual field stimuli. The identification of sex-hormone derivatives as predictors of cognitive behaviours is of importance. The finding suggests that there is a fundamental difference between the up-surge and decline of hormonal concentrations where previous studies typically assume all points near the peak of a hormonal surge are the same. How contradictory findings in sex-hormone research may have come about are discussed.

## Introduction

Changes in female human behaviour as a consequence of hormonal fluctuation over the menstrual cycle are a richly investigated area of science [1], [2] providing fascinating findings about sex hormone mediated behaviour. One prolific finding is that the sex hormones progesterone and estrogen have substantial effects on a females' performance of spatial tasks, particularly the mental rotations task [3]–[6]. Spatial tasks often involve the manipulation of mental images and it is often the case that males are reported to be better than females demonstrated by studies on both rodents [7] and humans [8]–[12]. Other findings include hormone related changes in memory [13], [14], verbal abilities [15] and facial recognition [5], [16] to name but a few. However, many of these findings show large inconsistencies between studies. This may be due in part, to the incomplete representation that typical two-time-point repeated measures studies give of the female hormone profile which fluctuates rapidly. The following article presents a longitudinal study which exploits an established spatial recognition paradigm [5], [17] to assess how a rich data-set (12 time points) compares to data from the typical two-time-point repeated measures design.

The “textbook” natural menstrual cycle of the average female (aged between 20 and 40) lasts for approximately 28 days. In reality, however, a normal cycle can last from 25–35 days, can vary intra-individually, and shortens noticeably after the age of 35 [18]. Researchers, when studying hormones-behaviour interaction, often compare performance during the follicular and luteal phases of the menstrual cycle. This is because these phases are characteristically distinct in their distributions of the sex-hormones estrogen and progesterone. Progesterone levels during the follicular phase are very low and unchanging at a baseline level whilst estrogen concentrations peak sharply towards the end of the phase. The follicular phase is characteristically variable in length, depending on the arrival of the inconstant ovulatory period. The luteal phase is more constant at 14 days [19], [20] and is characterized by the rise and fall of both progesterone and estrogen, the peak of which, on the average cycle coincides with approximately day 21. The majority of studies attempt to capture data at the peak sex-hormone period of the luteal phase and compare it to early periods of the cycle in the follicular phase. This approach provides maximal differences in hormone concentrations [5]. Thereby the approach allows researchers to examine the impact of sex-hormones on behaviour during periods with lowest and highest hormone profile. However, this method also artificially reduces the whole menstrual cycle to only two time points. This approach belies the significant variance in hormones over the complete cycle. Furthermore it is notoriously hard to capture the peak of the progesterone and estrogen during the luteal phase without exact knowledge of when ovulation occurred, necessitating further hormone testing. As far as we know only two studies have applied longitudinal paradigms over a period of 1–2 menstrual cycles.

Courvoisier et al [4] noted a linear relationship between performance on a mental rotation task and progesterone during the beginning of an eight week trial. Furthermore a quadratic relationship was found between performance and both estrogen and testosterone concentrations during the same time period. By the end of the study this relationship was no longer apparent. The authors suggested that the loss of a relationship at the end of the study signified that the impact of hormones become less relevant to the processing of the stimuli after repeated exposure. These data imply complex relationships between performance and hormone concentrations which may be obscured by repetitive training. Hausmann et al [17] showed correlations between progesterone and outcome measures varied across the cycle on a lateralised figural comparison task. The authors did not report a replication of previous work by showing direct comparisons between peak and low progesterone phases. This was perhaps due to the low power of the design for this comparison (n = 12) precluding replication of the basic finding. The present study attempts to resolve these findings into a single model which will explain the variance observed across the cycle.

The commonly held view is that sex-hormones exacerbate pre-existing, sexually dimorphic cognitive abilities. For example spatial tasks which exhibit large sex-related differences in performance such as the mental rotations task [21] are subject to large fluctuations over the female menstrual cycle [3], [5], [22]–[25]. The susceptibility of these tasks to hormonal modulation has often been attributed to their lateralised nature i.e., one hemisphere of the brain is highly specialised at completing the task. Changes in lateralisation of performance over the menstrual cycle has been extensively studied with both behavioural [5], [26]–[29] and fMRI paradigms [30]–[32]. In both lexical decision and figural comparison tasks performance in a groups of participants was less lateralised during the luteal phase than during the early-follicular phase [5], [17]. The finding of this pattern prompted the development of the progesterone-mediated interhemispheric decoupling (PMID) hypothesis [5] which provides an insightful and compelling hypothesis for explaining dynamic changes in cognition across the cycle. This hypothesis is based upon progesterone modulating pre-existing functional cerebral asymmetries. More specifically the hypothesis posits that increased progesterone reduces asymmetry which leads to increased or decreased performance depending upon what was the typical hemispheric pattern of lateralization. The hypothesis was updated subsequently to include proposals as to the asymmetrical effects of estrogen on interhemispheric interaction [29], [33].

Cahill identified a number of common misconceptions made within the scientific community regarding sex-related differences [1]. These misconceptions include: i) Sex-related differences in the brain are small, unreliable and result from a few extreme cases. ii) All differences in behaviour can be explained *entirely* by hormones. iii) Absence of sex-related difference in a given behaviour indicates that there is not any sex-related difference in the neural substrates for that behaviour. These are comprehensively addressed by Cahill as fallacy but he acknowledges however that the belief that sex-based behavioural differences are “small and unreliable” has “some evidence for, and some evidence against”. Central to the debate is whether or not certain sex-related differences exist [1], [34]–[37] and whether they are driven by estrogen or progesterone [38], [39], whether or not lateralisation changes over the menstrual cycle as a result of modulation of the left or right hemisphere and whether this due to a low or high concentration of hormones [30], [40].

One explanation for the contradictory findings could be attributed to subtle differences within the tasks and the degree to which the neural networks sub-serving that task are sensitive to sex-hormone modulation. Another, perhaps more likely, explanation for the contradictory findings may lie within the experimental design employed. Typically hormone and behaviour studies are almost exclusively dependent upon categorical comparisons between follicular and luteal phases. Within this design there is an inherent and perhaps erroneous assumption that all data points within these two phases are more or less equal. For example the ratio between estrogen and progesterone is not equally distributed across the phases and behaviour could be a consequence of the interaction between hormone concentrations rather than the independent levels. Additionally, just as the first hour of the morning as one awakens is not qualitatively the same as the last hour of the evening prior to sleep it may be that the surge and decline of sex-hormones (the derivative) lead to qualitatively different effects. Finally, obtaining results at the critical time point during categorical studies is exceptionally difficult and prone to error due to mistakes in self-reported menstrual cycle length [41]. In an attempt to circumvent all these issues we implemented a repeated measures design in which normal cycling female participants were tested 12 times every 3–4 days using the lateralized figural comparison task. This task was selected as it was the task shown to have the greatest effect of lateralization in the key paper proposing the PMID hypothesis [5]. Participants undertook the task whilst having eye movements measured using an eye tracker and salivary assays of progesterone and estrogen were taken on each occasion. Hormonal data was used to model behavioural outcomes of accuracy rates and reaction times. The first hypothesis was that salivary estrogen and progesterone concentration data can be used to effectively model accuracy rates and reaction times in the lateralized figural comparison task. The research questions surrounding this hypothesis is whether simply categorizing periods of the cycle into low and high hormone phases is a sufficiently sensitive approach. Or whether accounting for direction and gradient of hormone change and/or ratio is more effective. The second hypothesis was that using salivary estrogen and progesterone concentration data to model accuracy rates and reaction times will be more effective for stimuli presented in the right visual field compared to the left. The second hypothesis is based on the premise that the stimuli presented in the left visual field are presented to the specialized hemisphere for the task and thus less likely to be susceptible to the influence of sex hormones, or to have compensatory mechanisms in place [42]. Brain regions specialized to a key task with no benefit of being attuned to the menstrual cycle should have developed computational processing mechanisms that operate in spite of sex-hormone modulators. Consequentially, one would anticipate the effects of variance in sex-hormone concentrations to be more closely linked to the variability of behavioural measures in non-specialized neural circuits than in specialized neural circuits. Therefore, in this study using laterally presented figural comparisons, we would anticipate stimuli presented in the right visual field (to the non-specialized left hemisphere) to show greatest sensitivity to sex-hormone dynamics.

## Materials and Methods

The study was approved by the University of Surrey Ethics Committee and informed written consent was signed prior to participation.

### Participants

Twenty one naturally cycling, right-handed females (Age *M* = 20.95, 4.40*s.d.*, range 18–35) with normal or normal-to-corrected vision and no history of psychiatric illness were recruited for this study. All participants reported not having taken any hormone contraception in the year proceeding the study. One participant was excluded post-data collection due to low progesterone values across 11 of 12 sessions therefore *n* = 20. Women likely to have anovulatory cycles were excluded at pre-selection, i.e., if they had missed a period during the previous 12 months or reported a history of abnormal menstrual cycles. Additional criteria for exclusion included reported use of recreational drugs, heavy smoking (>10cigarettes per day) or heavy drinking (>20 units per week). Participants were asked to not drink or smoke heavily throughout the duration of the study and were asked to report immediately if they experienced anything unusual with their cycle or any high levels of stress.

Participants were a mixture of post and under graduate students, university staff and professionals living in the local vicinity of the University of Surrey. Remuneration of £100 was given to the participants upon completing the entire study.

### Experimental design

The study was a longitudinal repeated-measures design consisting of one screening session and 12 1 hour experimental sessions. The sessions were scheduled to be within 2–3 days of each other or at the closest practical time (gap in days between sessions *M* = 2.88+/−1.44). Participants were not required to begin the study at any particular stage of their cycle.

### Experimental procedure

Mean duration of the study was 33 days ranging between 28 and 48 days. Time of day effects were minimized by ensuring that each participant was scheduled within the same 2 hr time window on every visit. All participants agreed to and confirmed to ‘nil by mouth’ except water for the two hours prior to each testing session. All participants were aware of the study aims and the study was conducted by a similarly aged female researcher.

During each of the twelve experimental sessions participants followed the same routine during which five saliva samples were collected. First participants completed the Profile of Mood States (POMS) questionnaire whilst providing the first saliva sample. Subsequently participants carried out a 10 trial practice block after which they provided a second saliva sample. Participants then completed the first block of trials (120 trials; approximately 15 minutes) followed by a 2–5 minute rest period the length of which was dictated by the participant. During this rest period participants provided their third saliva sample prior to completing the final block (120 trials; ≈15 minutes). Upon completion of the second block participants provided the fourth and fifth saliva samples with approximately 15 minutes between samples. In an attempt to downplay menstrual cycle threat mood and performance were never referred to as changing over the menstrual cycle.

### Materials

#### Hormone Assessment

Hormones data was gained from five saliva samples taken during the course of each session. Participants passively drooled saliva down straws into collection tubes. Five samples were taken over the duration of the session and then pooled in order to get an average hormone concentration which should represent a more precise representation of the daily hormone concentration. In order for accurate measurement of hormones participants were asked to have had nil by mouth except water and to have not brushed their teeth in the two hours proceeding the session. The samples were transferred to a freezer (−20°C) until the end of the study when they were sent by overnight courier for radioimmunoassay by IBL-international in Hamburg, Germany (http://www.ibl-international.com). The sensitivity of the progesterone analysis was 2.6 pg/mL with a mean intra-assay variation of 3.4% and inter-assay variation of 9.9%. The sensitivity of the estradiol assay was 0.3 pg/mL with a mean intra-assay variation of 9.9% and inter-assay variation of 11.6%. Hormone data is available upon request.

#### Stimuli

The stimuli set consisted of 120 irregular black polygons on white backgrounds. The stimuli were identical to those used by Hausmann & Güntürkün [5]. Each polygon had at least 8-sides. Stimuli were provided by Dr. Markus Hausmann.

#### Questionnaires

The POMS and International Personality Inventory (IPIP) (www.ipip.com) questionnaires were administered. The POMS is a 65 item mood inventory which asks participants to rate on a 5-point Likert scale how much they relate to each item, an adjective such as ‘sad’, ‘cheerful’ or ‘restless’, at the time of taking the questionnaire. The POMS is capable of measuring transient, fluctuating and subtle changes in the factors tension-anxiety, anger-hostility, confusion-bewilderment, depression-dejection, fatigue-inertia and vigour-activity [43]. Participants completed the PoMS as a paper questionnaire at the beginning of every session prior to starting the experiment. The participants were instructed to answer the questionnaire without thinking for too long on their answers.

The IPIP is a well validated measure of personality which has five factors; agreeableness, extraversion, emotional stability, conscientiousness and intellect [44].

#### Eye-tracking

Eye tracking was conducted using a Tobii ×60 eye tracker (Data rate = 60 Hz) in a lit room. Participants were seated 60 cm from the PC screen, resting their chin on a chin rest. Lateralized stimuli (30 mm^2^) were presented 7.6° from centre (6.2° = inside edge; 9.0° = outside edge of stimulus). Participants were instructed to keep their head positioned on the chin rest and to maintain their gaze centrally fixated throughout the experiment.

#### Paradigm Design

**Figure 1:**
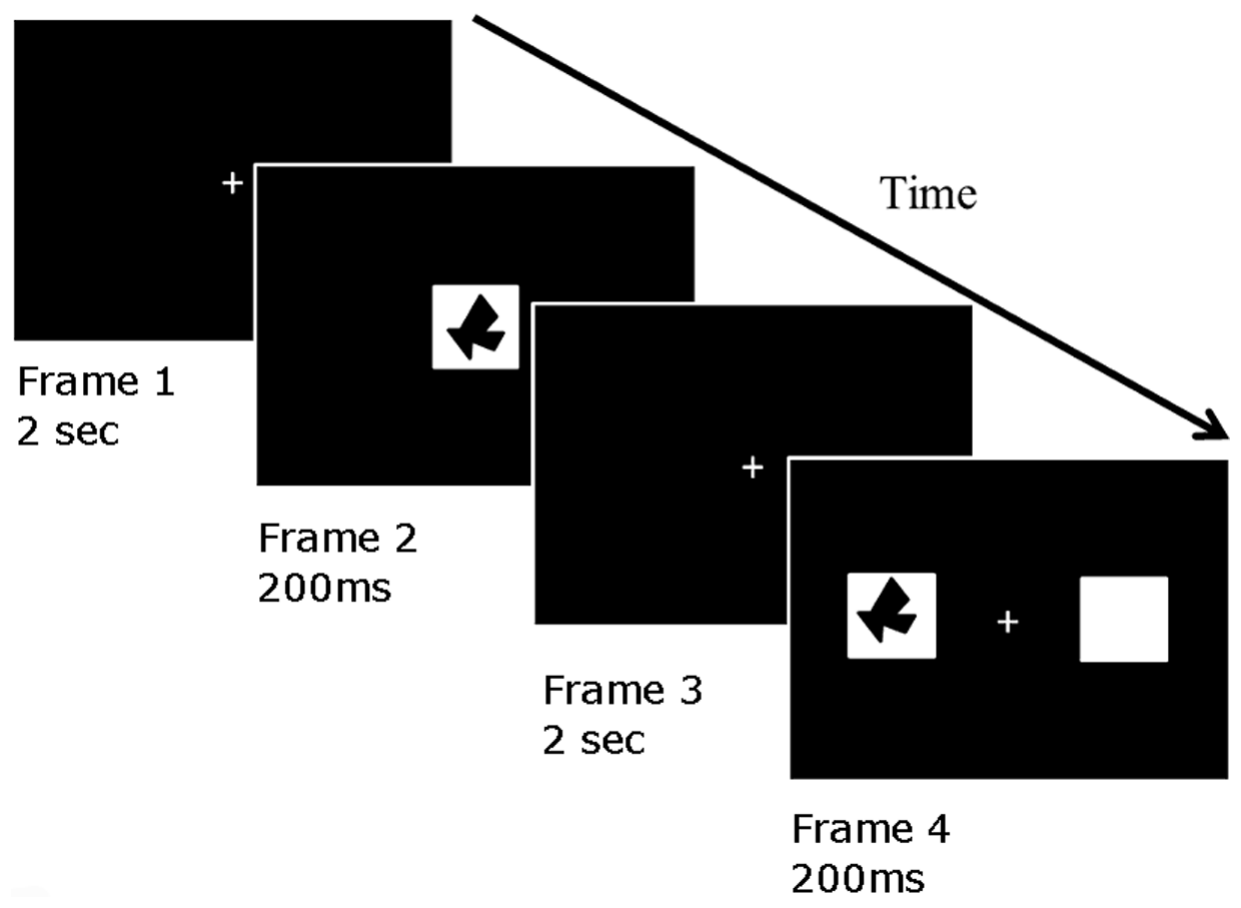
A cartoon showing the figural comparison, match-to-sample task. A trial commences with an inter-trial interval (2 sec) during which a white central fixation cross is shown on a black background. A centrally presented black polygon is presented for 200 ms followed again by white central fixation cross on a black background (2 sec). Subsequently two white squares are presented to the left and right of the crosshair. In one of the two squares a polygon is presented. The participant must indicate whether the laterally presented stimulus was the ‘same’ or a ‘different’ polygon to that presented previously.

We utilize a *‘match to sample’* task as shown in Figure 1. The paradigm essentially replicates the experiments conducted by Hausmann and colleagues [5], [17], [45]. Stimuli (white squares with black polygons) were presented on a black background using Presentation version 0.71 (Neurobehavioural Systems). Each session consisted of ten test trials, and two blocks of 120 trials. Participants changed hands for giving response between blocks in a counterbalanced manner. A single trial consists of four frames. Frame one was a white crosshair centrally presented for 2 seconds. Frame two was a single stimulus (polygon) centrally presented for 200 ms. Frame three was a white crosshair centrally presented for 2 seconds. Frame four consisted two simultaneous, laterally presented stimuli for 200 ms (see above for positioning). One stimulus was an empty white box, the other contained a polygon which was either the same (50% of trials) or different from the polygon shown in frame one. Left and right presentation of the polygon was counterbalanced. Frame four was a white crosshair centrally presented until the participant had indicated by button press on a computer mouse whether the two polygons shown in Frames 2 and 4 were the ‘same’ or ‘different’.

### Analysis

#### Pre-processing of eye tracking data

Eye tracking data was not available for 9 of 240 sessions due to a technical fault. A typical subject's eye tracking data can be seen in File S1 and the analysis technique described in the accompanying legend.

#### Processing of Behavioural trials

In addition to any trial removed due to eye movement trials, trials where the response time was greater than 2000 ms or less than 100 ms were also excluded. For both the left and right visual field presentation (LVF and RVF), we calculated the trimmed mean response times (RT) from the onset of the match stimulus. Accuracy (ACC) was calculated as the percentage of correct responses from trials included in the analysis. These values were entered into group statistical analysis. Data across sessions was not found to be normally distributed.

#### Assessing the effect of practice

We examined session effects on dependent variables using Friedman's ANOVA across the whole twelve sessions and over each set of four contiguous sessions. Summarized data is available upon request.

#### Categorical comparisons between high and low progesterone phases

The research question related to our first hypothesis questions the validity of using standard categorical comparisons between high and low hormone phase as typically conducted in research papers. To compare our data with previous studies, we selected the session with the highest progesterone measure across our twelve measures and took the sample ∼14 days (after normalizing data as described in the following section) pre/post the peak to represent a low progesterone follicular phase. Selected data points are shown on File S3. As all dependent variables were non-parametric, Wilcoxon signed rank tests were conducted to compare the effect of menstrual phase on accuracy rates and reaction times between progesterone high and low conditions.

#### Using estrogen and progesterone concentrations as predictors for modelling behavioural measures

Our first hypothesis assumes that a more sophisticated analysis taking into account the derivatives of the progesterone and estrogen concentrations may be effective in explaining variance in behaviour. It was assumed that all the experimental data is periodic in time and so could be fitted with trigonometric polynomials. The period (length of the cycle) is unknown for each individual and so the progesterone data was fitted with a third degree trigonometric polynomial in which the period was also assumed to be unknown using nonlinear least squares optimization. The period was restricted to being between 25 and 33 days unless the participant reported a shorter or longer cycle. The period was then fixed and the estrogen data together with the accuracy and response time data were each fitted with a third degree trigonometric polynomial using linear least squares optimization. To enable group comparison/fitting of a model using shared data the duration of the cycle must be made equal (normalized) across participants. Time was scaled to give a cycle length of 28 days and shifted so that the progesterone peak occurred at day 21 (the most robust measure). Average plots of all data against time were created to visualize changes common across the average cycle. All 12 data points were used for the hormone variables, but the first two sessions of each participant were excluded for the behavioural variables to counter possible learning effects occurring early in the experiment, resulting in 10 data points being used. A key aim of the study was to identify whether the hormone measures of salivary progesterone and estrogen values could be utilized to generate a model that would explain variance in behavioural performance. To investigate how the hormonal fluctuations may account for observed variance in behaviour we implemented a simple linear model with four predictor variables, progesterone (*P*), estrogen (*E*) and their derivatives *Pd* and *Ed*. An optimized model was identified for average and individual data and assessed using adjusted R^2^ (aR^2^) throughout this paper.

After ascertaining the model was sufficiently interesting to warrant further investigation we repeated the analysis but we excluded different hormone predictor variables on each occasion. This enabled us to ascertain each predictor variable's significance in regard to determining fit to each dependent variable. Secondarily to this we systematically utilized psychological variables from the POMs questionnaires to identify if the addition of a fifth psychological variable could improve our hormone based model.

#### Analysis of individual fit and value of model as predictor of behaviour

This analysis was conducted using models which include only hormone derived predictors *P*, *E*, *Pd* and *Ed*. Analysis was conducted on 19 of 20 data sets to derive an average model coefficient for *P*, *E*, *Pd* and *Ed*. The coefficients derived from the group of 19 were applied to the individual data set *left out* but the constant term was permitted to readjust to account for inter-individual differences in progesterone level etc. The aR^2^ was calculated to provide a metric of the predictive fit of the group to the individual. The analysis was iterative with each participant being utilized as the individual. The individual adjusted R^2^ fits of the optimized model (*P, E, Pd, Ed*) value for each dependent variable were entered into Spearman's correlation analyses to identify whether the fit of the model was a predictor of any individual differences between participants.

## Results

### Descriptives

The mean low and high of progesterone levels across participants were (M = 42.8 pg/ml+/−30.6 pg/ml *s.d.* for low and M = 148.9 pg/ml+/−81.6 pg/ml *s.d.* for high). Individual ranges varied from 35–427 pg/ml (range = 392) to 15–50 pg/ml (range = 35). The mean low and high of estrogen across participants was (Low M = 3.2 pg/ml+/−1.3 pg/ml *s.d.* for low and M = 5.6 pg/ml+/−2.9 pg/ml *s.d.* for high). Individual ranges varied from 1.2–2.9 pg/ml (range 1.2) to 7.5–34 pg/ml (range = 26.5).

### Practice effects

Session effects were examined and identified and are fully presented in File S2. In summary, accuracy rates stabilized by session 3 yet response times fell more or less across the entire experimental period. This finding was the basis for removing sessions 1 and 2 from subsequent analysis.

### Categorical comparisons of high and low hormone phases

Table 1 shows the results of direct comparisons between conditions at both high and low progesterone. Table 1 shows no significant differences of lateralization were found although there was a trend towards significance (*p* = 0.07), with a large effect size (*r* = 0.41) when comparing RVF-ACC between high and low progesterone phases. File S3 indicates which data points were selected for these comparisons and shows boxplots of the data.

**Table 1:** Results of Wilcoxon signed rank tests between conditions, prog = progesterone.

Wilcoxon rank sum comparison	*Z*	*P*	*r*
(HIGH prog LVF-ACC) v (HIGH prog RVF-ACC)	−1.05	0.3	0.23
(LOW prog LVF-ACC) v (LOW prog RVF-ACC)	−0.02	0.99	0
(HIGH prog LVF-ACC) v (LOW prog LVF-ACC)	−0.63	0.53	0.14
(HIGH prog RVF-ACC) v (LOW prog RVF-ACC)	−1.83	0.07	0.41
(HIGH prog LVF-RT) v (HIGH prog RVF-RT)	−0.75	0.46	0.17
(LOW prog LVF-RT) v (LOW prog RVF-RT)	−0.04	0.97	0.01
(HIGH prog LVF-RT) v (LOW prog LVF-RT)	−1.34	0.18	0.3
(HIGH prog RVF-RT) v (LOW prog RVF-RT)	−1.12	0.26	0.25

The values of *Z*, *P* and the effect size *r*, are shown for each test. Data is shown graphically in Figure 3d.

**Figure 2:**
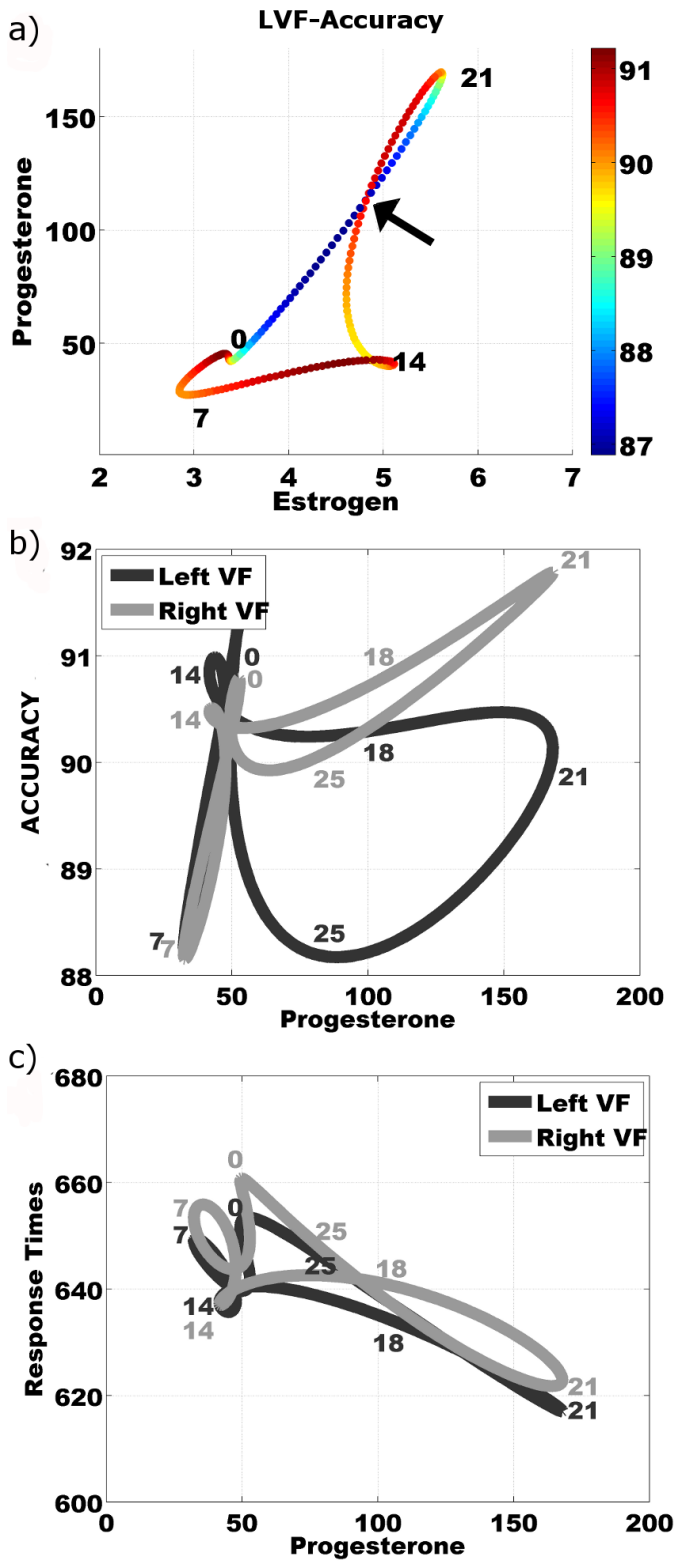
Phase plane plots of data averaged across participants. Numbers next to the line on the plot indicated the approximate day of a normalized 28 day cycle. Following a single plot allows one to follow data through a 28 day cycle. a) Phase plane plot of group's normalized (to 28 day) estrogen and progesterone saliva concentrations across the menstrual cycle. Colour indicates group's average Left Visual Field Accuracy (LVF-ACC) as percentage of trials where a correct response was given. The arrow indicates point where estrogen and progesterone values are equal but LVF-ACC is different. b) Group average LVF-ACC and RVF-ACC plotted against group average changes progesterone in a normalized 28 day cycle. c) Group average LVF-RT and RVF-RT plotted against group average changes progesterone in a normalized 28 day cycle.

Fluctuations in accuracy rates across the two visual fields were of particular interest as lateralization effects had been observed in previous studies. LVF-ACC is plotted on the phase plane plot in colour (Figure 2a). In this paper phase plane plots are used to show a cyclical function which is why the line is plotted as a closed loop. The loop plots the data over a repeating 28 day cycle. The arrow shows that at approximately days 18 and 25 the hormone concentrations are the same but the performance difference is at a maximum. Indeed the upsweep and down sweep of progesterone has very different performance characteristics which pivot at the peak. Figure 2b shows that on average there was little effect of visual field on accuracy other than a small bias for RVF stimuli during the progesterone down-sweep (day 21–25). There was however a considerable range in difference between left and right VF accuracy across the cycle (mean range = 12.2%, min range = 5.5%, max range = 21.0%). Yet variance was distributed equally across the menstrual cycle (See File S4 for detail). For alternative visualization we have plotted average curves of L/R VF-ACC and L/R VF-RT against progesterone concentration (Figures 2b and 2c). Figure 2c shows that response times show no lateralization effect at all. The ACC hemispheric difference noted in Figure 2a is more clearly observed in Figure 2b at a progesterone concentration of 80 pg/ml. We would like to draw attention to the fact that only the down-sweep of progesterone concentration days (21–28) shows differential effects. These effects are not in the hypothesized direction neither are they found at the ‘peak’ progesterone level. The up-sweep of progesterone (days 14–21) is relatively limited in generating change in behavioural outcomes.

### Estrogen and progesterone concentrations as predictors for modelling behavioural measures

Here we describe the results of using hormonal predictor variables to model behavioural outcome variables. Our basic hormonal predictors are the saliva hormone concentrations of estrogen (*E*) and progesterone (*P*) and the mathematical derivatives of the two measures (*Ed* and *Pd*). We first consider whether the two measures *P* and *E* alone can reliably determine the behavioural outcome variables ‘accuracy’ (ACC) and ‘response times’ (RT) to left visual field (LVF) and right visual field (RVF) stimuli. Before addressing this question, we note that the relative changes in progesterone and estrogen levels can be visualised by plotting one against the other over one cycle in a phase-plane plot. Since both these variables are assumed to be periodic, the trajectory in the phase-plane will be a closed loop (Figure 2a). The estrogen profile has two peaks, the first around day 14 and the second around day 21. The first peak is usually quite sharp, and so can easily be missed with data being collected only every 2–4 days.

The colour in Figure 2a indicates the LVF-ACC output variable at each point in the cycle. In the example phase-plane plot, there is one point at which the curve intersects itself (arrow), corresponding to two points in the cycle at which both the two individual hormone concentrations are the same. These two points occur just before and just after the progesterone peak. If the output variable depends only on the values of *P* and *E*, then its value on the two curves that intersect should be, at least approximately, the same. However, we can see from the plot in Figure 2a that this is not the case as the colours are very different on the two curves at the crossover point. In fact, we observe that some of the highest ACC scores are achieved on the up-sweep of progesterone, while the lowest ACC scores occur on the progesterone down sweep. Therefore, we conclude that the two hormone levels alone cannot explain the variation in the output variables over the cycle.

As the hormone levels cannot uniquely determine the output variables, we then considered whether *P* and *E* together with their derivatives *Pd* and *Ed* could be used to better fit the data. In particular, we considered a simple linear model of these four variables given by
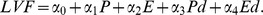



**Figure 3:**
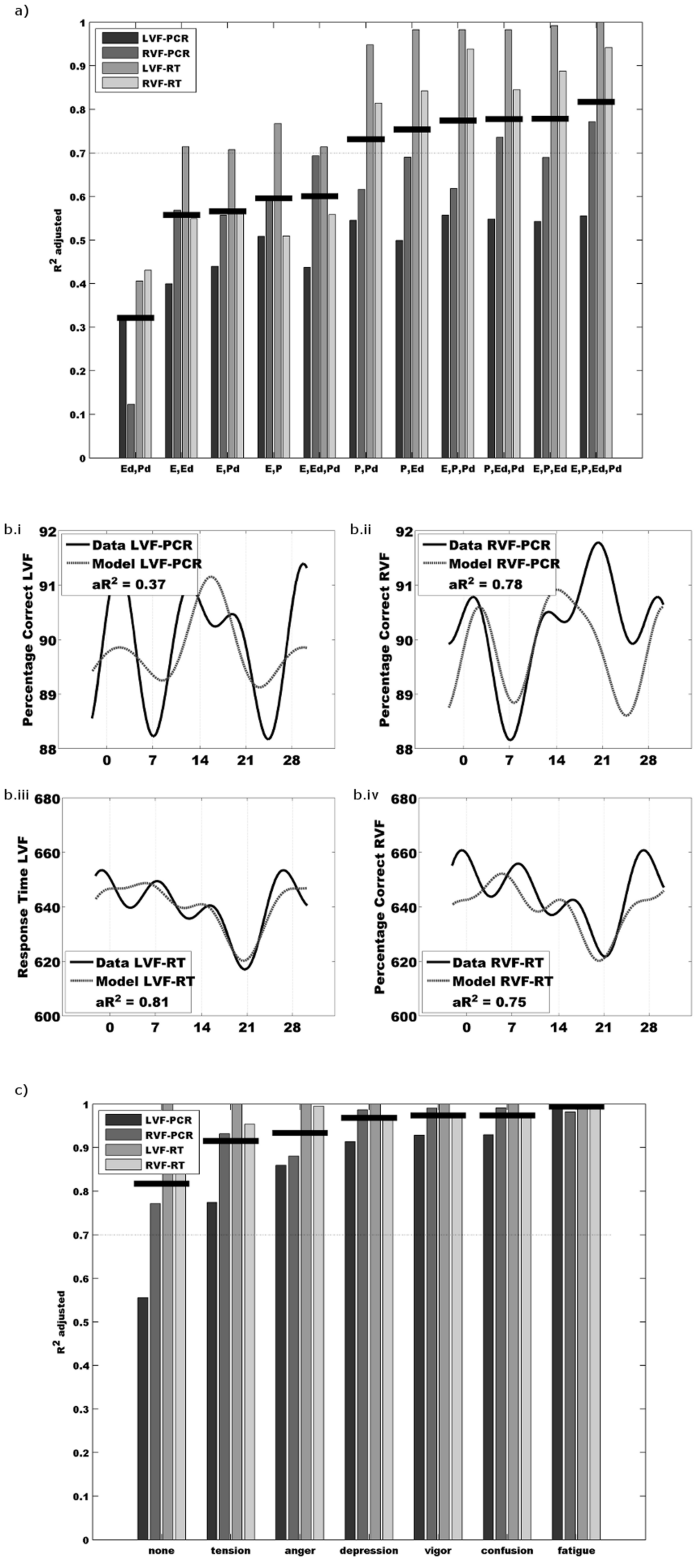
Result of modelling accuracy rates & reaction times with both hormonal and psychological variables. a) Impact of including different combinations of the salivary concentrations estrogen (*E*), progesterone (*P*) and their derivatives (*Ed* and *Pd*) upon prediction of behavioural outputs. Behavioural outputs include the ‘accuracy’ rates and ‘response times’ for the left and right visual. Fit measured as adjusted R^2^. Combinations plotted in ascending order of best mean fit (horizontal thick line) across the four output measures. b) A linear model was implemented using salivary concentrations of progesterone, estrogen and their derivatives in an attempt to explain the four behavioural outcomes. Each panel shows the group averaged data (unbroken line), the output of the model used to predict the data (dotted line) and the adjusted R^2^ value indicating the fit of the model to the data. i) Fit for LVF-ACC. ii) Fit for RVF-ACC. iii) Fit for LVF-RT. iv) Fit for RVF-RT. c) Impact of including different psychological variables of mood to the model. Behavioural outputs include the ‘accuracy’ rates and ‘response times’ for the left and right visual field. Fit measured as adjusted R^2^. Plotted in ascending order of, best mean fit across the four output measures.

A similar model for the other three output variables, but with different coefficients, was also used. Note that this model incorporates not only the hormone levels, but also their rate of change. Variations of this model utilizing all possible combinations of *P*, *E*, *Pd* and *Ed* were also tested (Figure 3a).

The best model fit was achieved by using all four predictors (Figure 3a). This model leads to a good fit for response times in both visual fields (LVF-RT *adjusted* R^2^ = 0.82; RVF-RT *adjusted* R^2^ = 0.75) and for accuracy rates of stimuli presented in the right visual field (RVF-ACC *adjusted* R^2^ = 0.78). However it failed to correctly predict accuracy for stimuli presented to the left visual field (LVF-ACC *adjusted* R^2^ = 0.37).

The fit was at its worst if either only the derivative variables *Pd* and *Ed* were included (Figure 3a) or if only information about one hormone (concentration and derivative) was provided. Including *P* and *Pd* in the model however led to a better model fit than using solely *E* and *Ed*. Thereby we can conclude that progesterone and its derivative appears to be the dominant, but not sole factor for predicting reaction times and RVF-ACC.

The output variable of LVF-ACC stands alone as not fitted by our approach. Good quality prediction of LVF-ACC is not attained under any combination of these four factors. In particular the model fails to account for variation in the follicular phase of the cycle (Figure 3.b.i). The LVF-ACC variable is also contrary to the other output variables in that it appears to be most linked to *Ed*, the predictor variable least able to predict the other three outcome variables. Adjusted R^2^ for LVF-ACC falls to less than 0.2 whenever *Ed* is omitted from the model.

### Testing Model Prediction

We subsequently investigate the impact of including a 5^th^, psychological predictor variable into our model in addition to the four hormonal predictors (Figure 3c). Mood measures, taken at each session, were added one factor at a time to observe whether they explained additional variance, particularly upon LVF-ACC. Factors tested were ‘tension’, ‘anger’, ‘confusion’, ‘depression’, ‘fatigue’ and ‘vigour’. Adding an additional predictor never reduced fit although impact on fit varied across output variables. The average fit (black horizontal line, Figure 3c) to the four output variables showed little variation with the highest being ‘anger’, (LVF-ACC *adjusted* R^2^ = 0.95; RVF-ACC *adjusted* R^2^ = 0.92; LVF –RT *adjusted* R^2^ = 0.94; RVF –RT *adjusted* R^2^ = 0.83). Only ‘tension’ stood out as being more ineffective than the others. The common component of test scores which most improved the model was a deviation from the mean test score around day 9 of the cycle.

Finally we sought to assess whether the model derived from the group data could be used to predict behavioural outcomes of a new participant given their hormonal data. Using a leave-one-out strategy we created a group model using *n*-1 participants and measured the fit of the model to the non-included participant. The approach did not attempt to be entirely predictive as the model was optimized to fit on the *y* axis to account for the individual's mean reaction time/accuracy rate and the scale of the range of variance. However the shape of the curves derived from the group model was held constant. The adjusted R^2^ values for each behavioural output all fall well below threshold, (LVF-ACC *adjusted* R^2^ = 0.08; RVF-ACC *adjusted* R^2^ = 0.45; LVF-RT *adjusted* R^2^ = 0.42; RVF-RT *adjusted* R^2^ = 0.30). LVF-ACC can be noted to continue to be a case apart from the other three behavioural output measures. LVF-ACC consistently fails to be predicted by hormone levels to the same degree as RVF-ACC and response times in both visual field conditions. We conclude that a model generated using a set of individuals is unable to predict changes in behaviour to novel data not included in the model development.

## Discussion

We implemented a lateralized figural comparison task in a multiple repeated measures design whilst measuring salivary concentrations of progesterone and estrogen in human females exhibiting a normal menstrual cycle. The aim of the experiment was to investigate the relationship between changes in hormone balance upon basic behavioural outputs in this visuo-spatial task.

### Categorical comparisons between high and low progesterone phases

Categorical comparisons between high and low progesterone phases are the standard manner in which to conduct studies of the impact of ovarian hormones upon behaviour [46]. Often findings are contradictory and one potential reason may be due to the large variability in menstrual cycles across women leading to inaccurate sampling. Due to the regular sampling of our cohort we are able to more accurately select time points for a similar comparison as to that made in other studies. Our estimate of the progesterone peak is accurate to within two days and we also know which side of the peak from which we sample. We account for cycle duration and sample the low progesterone phase at ¼ of the way through the individual's cycle. Due to the power of our study and tight control of anticipatory eye movements and session effects we were confident that our study would enable us to definitively demonstrate the effect of menstrual phase upon changes in accuracy in this spatial task. We found a ‘trend’ (p = 0.07) with a large effect size (r = 0.41) for RVF-ACC rates to be higher during luteal phase compared to follicular phase as observed previously [5]. We conclude there is a small but reliable effect of menstrual phase upon RVF-ACC rates.

### Estrogen and progesterone concentrations as predictors for modelling behavioural measures

The first hypothesis was that salivary progesterone and estrogen concentration data can be used to effectively model accuracy rates and reaction times in the lateralized figural comparison task. We showed this can be done and additionally that the derivative of the two sex-hormones are important variables for modelling behaviour. Progesterone is a more powerful factor for fitting behaviour to response times (RT) and accuracy (ACC) rates in general. Estrogen however showed a greater role in fitting ACC rates to stimuli shown in the left visual field (LVF) thereby projecting to the brain hemisphere specialized for this task. Although we could produce a satisfactory model that explained behavioural outcomes at group level, the group level models were not predictive of individual behaviour.

### The value of hormonal derivatives as predictors for behavioural measures

This model demonstrates for the first time that the gradient and direction of change in hormone concentration, i.e., the derivative, is a useful measure in determining a cognitive behavioural readout. The removal of the estrogen derivative has the most detrimental effect upon our model over and above the removal of estrogen or progesterone raw concentration values alone. This is in great part due to the impact the estrogen derivative has on improving LVF-ACC performance predictions over and above the other three variables (LVF-RT, RVF-CR, RVF-RT). On the whole progesterone saliva concentration appears to be the most informative of our hormonal measures for modelling behaviour. The removal of the progesterone derivative from the model has a greater impact than removing estrogen (as long as the estrogen derivative is retained). The derivative therefore is of notable consequence when modelling behaviour. This finding is of great interest because it demonstrates why previous studies may find it so difficult to replicate one another. It demonstrates that the rate and direction of the rise/fall of the hormones being measured may well be critical to the understanding of the snapshot which has been captured. Typically it has been assumed that all data points around ‘peak progesterone’ can be categorized together as ‘luteal’ or ‘high progesterone’ and compared categorically to those in the follicular phase or ‘low progesterone’. What is demonstrated here is that the progesterone-surge and progesterone-decline are two very different categories. A consequence of this is that if research groups are inadvertently using participant selection criteria biased towards the capture of one subsection of the luteal phase we would expect mixed results appearing in the literature. These findings also harmonize another debated aspect of hormone research which concerns the question whether estrogen or progesterone is most relevant to behaviour change. The differential effects observed for the *E* and *P* derivatives in the present study suggest different functional roles for *E* and *P* in the modulation of cognitive function. Specifically we propose that *E* facilitates specialised (i.e. lateralised) circuits while *P* affects predominately none-specialised circuits.

In summary, utilizing hormonal measures of salivary hormone concentrations and their derivatives proved to be a powerful predictor of individual's behavioural outcomes with the exception of the accuracy rate of stimuli presented in the left visual field (LVF-ACC), i.e., the hemisphere specialized for spatial awareness. Psychological factors which showed variance around day nine improved the model's fit for individual participants in particular for LVF-ACC.

### Potential third biological predictor of behaviour

Estrogen and progesterone concentrations and derivative values are biological variables which allow successful modelling of behavioural outcomes in a simple lateralized visuo-spatial match to sample task (Figure 3). Modelling of the LVF-ACC behavioural outcomes was not successful using biological variables alone (Figure 3b.i). Using mood measures it may be noted that very high fit can be generated and one can note a specific shape to the curves generating the best fits. Those factors which show variance across the cycle which contains a strong change around day nine, ‘anger’ (increase), ‘depression’ (increase) and ‘vigour’ (decrease) seem to describe the most accurate model. We can therefore suggest that a biological mechanism, hormone or neurotransmitter plausibly linked to these three moods may well be the third predictor required for predicting behaviour across the menstrual cycle.

### Effect of lateral presentation upon model

The second hypothesis was that using salivary estrogen and progesterone concentration data to model ACC rates and RTs will be more effective for stimuli presented in the right visual field compared to the left. This was demonstrated to be the case specifically for ACC rates but not RTs. Our hypothesis was that neuronal populations that had evolved for specialized processing would demonstrate counter mechanisms to the global modulation of GABA and glutamatergic processing by the hormones estrogen and progesterone. The premise of this hypothesis was that using hormones to change behaviour by modulating GABA and glutamate was a useful evolutionary mechanism, yet it would require a counter mechanism to ensure not all neural systems were affected [42]. Consequently, sensitivity within neural systems to these hormones would require the ability to be attenuated. We tested this using a lateralized visuo-spatial task assuming that the right hemisphere would be specialized to the task and the left hemisphere would not. Therefore we anticipated progesterone and estrogen to be effective predictors of behaviour for stimuli presented in the right visual field but not the left. We found that the behavioural variables could not be reliably predicted using only the two hormone concentrations, but that a simple linear model including progesterone, estrogen and their derivatives provided an excellent fit for behavioural data at individual and group level for all behavioural measures (response times and accuracy) except the accuracy rates of the left visual field stimuli (LVF-ACC). The insensitivity to hormone fluctuations within the specialized network (LVF-ACC) supports the hypothesis that a mechanism exists to counteract the impact of estrogen and progesterone within specialized neural circuits [42]. Presumably this enables evolution to utilize the hormonal cycle to allow some aspect of behaviour to be driven by hormonal changes whilst simultaneously preventing others from doing so in a maladaptive manner.

### Effect of lateral presentation upon behaviour

After normalizing data we were able to extract each participant's session equivalent to day 7 and day 21 (File S3), thereby assessing lateralization effects at high and low progesterone as is the common approach. We observed no effect of lateral presentation at either high or low progesterone. Because each participant's equivalent of day 7 or day 21 was at a different session in their training regime, we cannot discount the possibility that training effects may have masked the effects of lateralisation. However, women were equally spread in terms of their starting point of the study relative to menstrual phase therefore the impact of the session selected would lead to noise not bias in terms of training effects. Taking the power of the data and the low effect sizes observed however we must conclude that lateralization effects are not observed in the lateralized spatial figural comparison task within a menstrual phase after removing anticipatory eye-saccades and removing bias due to session effects.

Contrary to previous studies we have found no evidence of differences in response times or accuracy rates with regard to the lateralized presentation of stimuli during a particular menstrual phase. We suggest that the inter and intra individual variance may be responsible for the mixed findings across many previous studies investigating effects of lateralization in female subjects. Given the experimental power and experimental controls we consider our findings to be reliable and robust. We have identified an effect of learning across sessions in terms of response times. Additionally we have found effects of learning on accuracy rates across the first four sessions, albeit the third and fourth sessions do not markedly differ from the subsequent sessions. As alluded to above, it may be that effects of lateral presentation are only apparent in the early sessions (1–4) and after learning, effects of lateral presentation are lost. We do not observe this at all but it must be clearly acknowledged that each participant's ‘early sessions’ were spread across the whole cycle. Therefore it is possible that if such learning trials were conducted at a comparable time point across all participants, effects of lateral presentation might be identified. Most studies do of course have this structure as they utilize repeated measures designs with only two time points, collecting data during follicular and luteal phases counterbalanced for order. An interaction between testing-order and cycle phase has been demonstrated with a stronger decrease in performance being associated with the first session falling in the luteal phase [5]. Finally, our method of retrospectively assigning participants to phase groups based on actual peak hormone levels has the benefit of being more accurate than relying on potentially unreliable self-reports of menstrual status to assign groups [47]. We must therefore conclude that lateralization effects are not observed in the lateralized spatial figural comparison task within a specific menstrual phase.

### Limitations and potential mis-interpretations

#### Stereotype threat

Stereotype threat is when an individual feels at risk of confirming a negative stereotype about one's own group [48] When negative stereotypes are made salient this risk increases, which in turn has a negative impact on cognition [49]. Menstruation has long been associated with debilitated function [50]. Wister et al [48] found that activating menstrual cycle threat had a debilitating effect upon women's performance the closer they got to menstruation even after positive primes regarding menstruation. This occurred despite the women reporting that they did not hold strong beliefs about their menstrual cycle. Therefore, it is possible that if menstruation is made salient to women and they are aware of negative stereotypes surrounding menstruation then this may have a negative impact upon their cognition during cognitive tasks. Our study required self-report data about menstruation from the individual thereby making the individual aware of the purpose of the study and thus making the threat salient and a potential confound.

#### Application to wider population

Tightly controlled laboratory studies are excellent for examining the complexity of behaviour yet one must be wary of over generalization. We utilize a Western, Educated, Industrialized, Rich, Democratic (WEIRD) demographic [51] with very specific selection criteria. It is necessary to question the broader significance of a study that so tightly selects participants. Infrequent cycles, anovulatory cycles, and long periods of pregnancy and/or breastfeeding are very common in reproductively aged women all over the world. To understand the impact of hormones on behaviour across society it would be more effective to utilize technology to deliver experimental protocols *en masse* across cultures to examine both the aforementioned factors as well as other factors such as diet, sunlight, socio-economic class etc. The ubiquity of smartphones and software to develop experiments (www.psyapp.co.uk) will facilitate this kind of macro level study [52]. This study demonstrates that the gradient and direction of hormonal changes of concentration influences behaviour in a simple cognitive visual-matching task. This is a mechanism that should be considered to perhaps be occurring in any behaviour/hormone study.

#### Seeking a positive evolutionary rationale

We do not expound a positive evolutionary rationale for this cognitive sensitivity to hormones in basic cognition. We subscribe to De Vries [42] general idea that neural circuits experience selective pressure to compensate against the modulatory effects of sex-hormones rather than evolve mechanisms to respond to them. That is to say sex-hormones are pre-requisite to successful reproduction and the rest of the organism must evolve around them and in spite of them. Observation of ‘effects of sex-hormones upon behaviour’ by researchers is the observation of neural circuit performance which ‘have not’ developed compensatory mechanisms. Neural circuits will ‘not’ develop compensatory mechanisms for one of two reasons. 1) A positive selection pressure to retain sensitivity i.e., modulating sexual arousal to salient cues regarding partner selectivity. 2) An absence of selection pressure upon a circuit. For example a circuit may not be utilized typically for a task except in experimental studies of laterality thereby it will not have experienced selection pressure. We suggest brief lateral presentation of shapes to the non-dominant hemisphere shows greater effects due to neural circuits ‘not’ having been tuned by evolutionary selective pressures to compensate against sex-hormones.

## Conclusions

The sex-hormones estrogen and progesterone are both linked to response times and accuracy rates in a simple figural comparison task. The derivatives of these two measures are important predictors of behaviour in addition to the raw sex-hormone concentration. Differentiating between upsurges and declines of hormonal changes is necessary to predict behaviour. The findings suggest neural circuits specialized for particular tasks are attenuated in regard to their sensitivity to sex-hormones. Non-specialized circuits appear more sensitive to sex-hormones, specifically progesterone. Specialized circuits, whilst attenuated in their modulation, are more influenced by estrogen.

## Supporting Information

File S1 **Eye tracking data analysis methods.**
(DOCX)

File S2 **Effects of session (independent of cycle phase) analysis.**
(DOCX)

File S3 **Categorical comparisons of high and low hormone phasesa.** Analysis imitating that conducted in standard two session repeated measures experimental designs after post-hoc selection of most appropriate time points.
(DOCX)

File S4 **Visual field advantage over the menstrual cycle.**
(DOCX)
