# Supplementary material for: The Importance of the Derivative in Sex-Hormone Cycles: A Reason Why Behavioural Measures in Sex-Hormone Studies Are So Mercurial
Source: PLoS One. 2014 Nov 26;9(11):e111891. doi: 10.1371/journal.pone.0111891 (PMC4245079; doi:10.1371/journal.pone.0111891)
Supplement: File S1 — Eye tracking data analysis methods. (DOCX) [file pone.0111891.s001.docx]

Eye tracking data was not available for 9 of 240 sessions due to a technical fault. However sessions without eye tracking data are spread evenly across subjects and across the cycle. All data was analysed in MATLAB (The Mathworks Inc., Natick, MA). The *x* and *y* coordinates for eye movements were extracted for each trial. All trials during which gaze had significantly moved towards the laterally presented stimulus or where the participants blinked during stimulus presentation were removed prior to further analysis. A semi-automated approach to thresholding for gaze shift was utilized. Each session’s eye tracking data was visually inspected post-automated sorting to ensure validity. Threshold was primarily calculated (72% of sessions) against 32 calibration trials presented at the beginning of each session. During calibration participants made saccades to the laterally presented stimulus from the central focal point as opposed to maintaining central fixation. Calibration trials did not always accurately represent an average lateralized saccade. This may have been due to the relatively low number of calibration trials collected or because some participants did not always make full saccades for each calibration trial, perhaps due to a perceived task irrelevance after multiple sessions. In such cases threshold was calculated in one of two manners. If it was common that the participant made a saccade that followed the onset of the lateralized stimulus, this was used to calibrate the size of the saccade (18% of sessions). If the participant maintained a very steady gaze and practically never moved their eyes (10% of sessions) a conservative manual threshold was added based upon other session’s data from that participant. Practically this final approach served mostly to remove trials in which the participant blinked. Trials were excluded from analysis if a shift greater than 3.8° towards the lateralized stimulus occurred during stimulus duration.

Trial exclusion rates due to eye movements and blinks were low for LVF (mean = 5.9, median = 2, trials/session) and RVF (mean = 7.9, median = 1, trials/session) stimuli in the first three sessions. This dropped slightly over the study period to LVF (mean = 2.5, median = 0, trials/session) and RVF (mean = 3.5, median = 0, trials/session) stimuli in the last three sessions. Trials excluded due to late responses were negligible for both LVF (mean = 1.8, median = 0 trials/session) and RVF (mean = 1.14, median = 0 trials/session) stimuli. The data suggests that participants were compliant to instruction to not move their eyes from central fixation, with a median of 3/120 trials being removed across all sessions where eye tracking data was available. Because participants were compliant with the task we included the 9 sessions for which eye tracking information was not available.


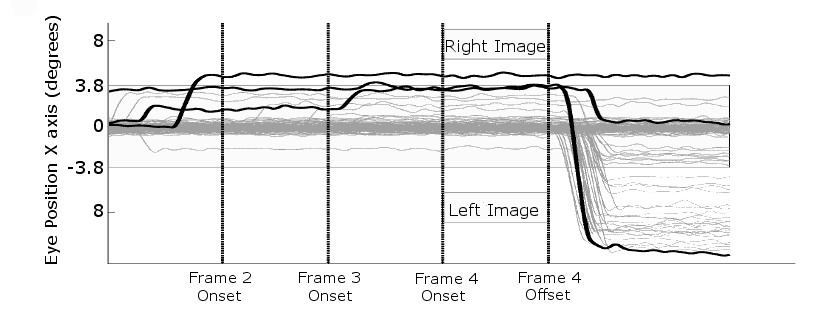


**Figure:** Traces from a typical sessions’ eye-tracking data is shown for trials with left visual field presentation of polygon. Traces of trials excluded due to passing threshold for eye gaze being off centre during Frame 3 are shown in bold.
