# Supplementary material for: The Importance of the Derivative in Sex-Hormone Cycles: A Reason Why Behavioural Measures in Sex-Hormone Studies Are So Mercurial
Source: PLoS One. 2014 Nov 26;9(11):e111891. doi: 10.1371/journal.pone.0111891 (PMC4245079; doi:10.1371/journal.pone.0111891)
Supplement: File S2 — Effects of session (independent of cycle phase) analysis. (DOCX) [file pone.0111891.s002.docx]

Session effects were examined across windows of four continuous sessions (1:4,2:5,3:6…9:12) using Friedman’s ANOVA to examine session effects upon performance with session as the single factor with four levels.


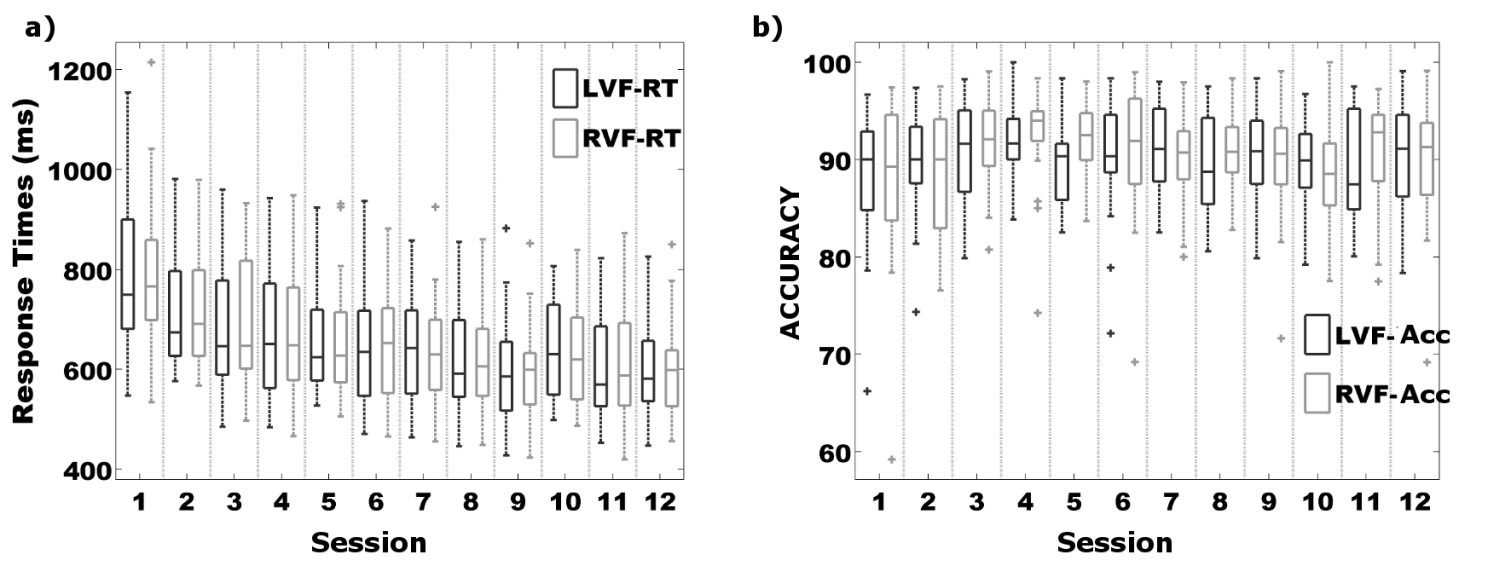


**Figure** Session specific effects for accuracy and response times. Both left and right visual field stimuli showed increasing accuracy across consecutive sessions in the earliest sessions. LVF-ACC showed greatest significant session effect between sessions 1 and 4 (*χ*^2^ (3, *N* = 20) = 9.69, *p<* 0.05) and RVF-ACC stimuli showed greatest session effect between sessions 2 and 5 (χ2 (3, *N* = 20) = 16.56, *p< 0.01*). Tests of sessions 3-6 and onwards showed no significant session effects. Session effects of decreasing response times were observed for both left and right visual field stimuli. Response times showed a significant session by session decrease (at threshold p < 0.05) across almost all possible sets of four continuous sessions. For RVF stimuli reaction times decreased significantly (p<0.01) for over half the time windows including time windows towards the end of the experiment e.g., sessions 8-11 (*χ*^2^ (3, *N* = 20) = 15.06, *p< 0.01*). In summary, accuracy rates stabilized by session 3 yet response times fell more or less across the entire experimental period. This finding was the basis for removing sessions 1 and 2 from subsequent analysis.
