# Supplementary material for: The Importance of the Derivative in Sex-Hormone Cycles: A Reason Why Behavioural Measures in Sex-Hormone Studies Are So Mercurial
Source: PLoS One. 2014 Nov 26;9(11):e111891. doi: 10.1371/journal.pone.0111891 (PMC4245079; doi:10.1371/journal.pone.0111891)
Supplement: File S3 — Categorical comparisons of high and low hormone phasesa. Analysis imitating that conducted in standard two session repeated measures experimental designs after post-hoc selection of most appropriate time points. (DOCX) [file pone.0111891.s003.docx]

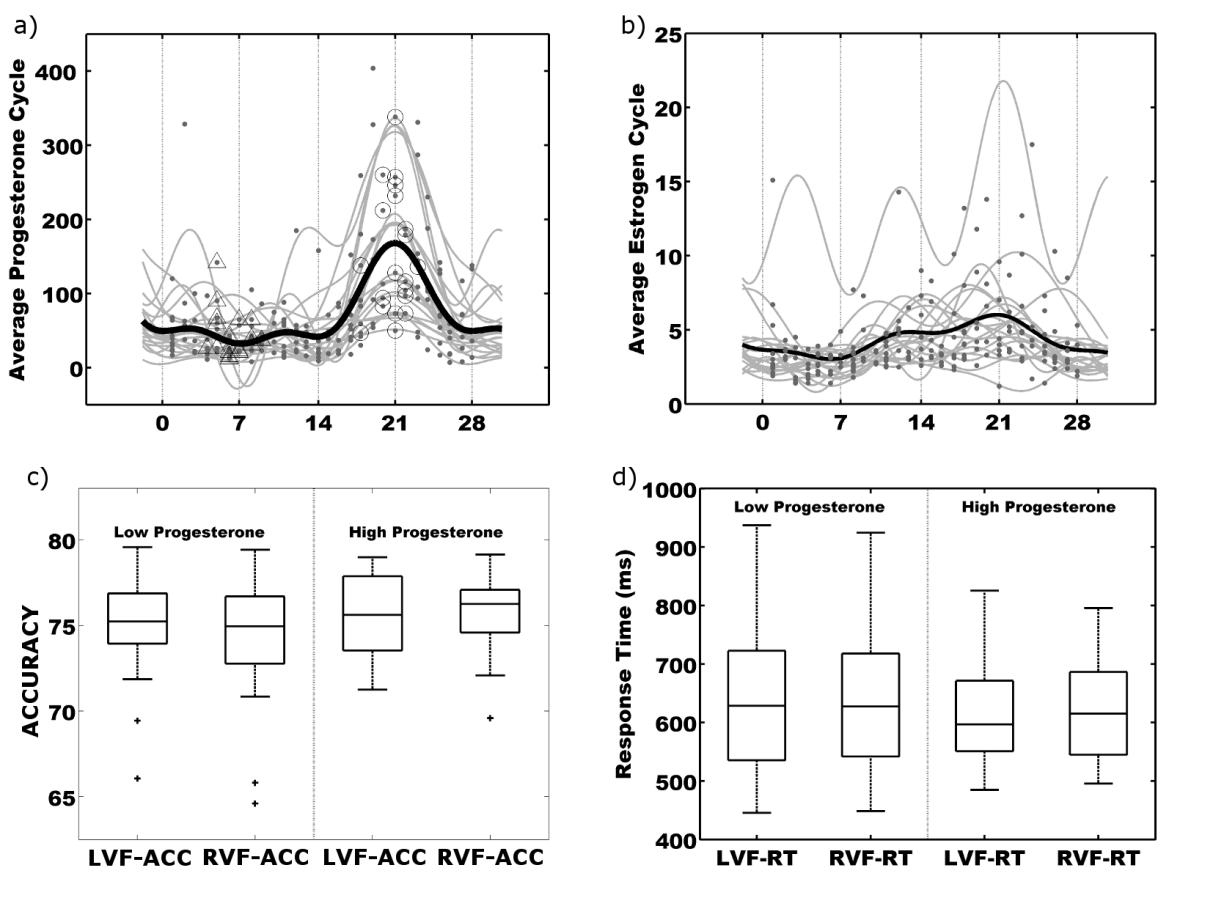


**Figure:** Categorical comparisons of high and low hormone phases. The thick line indicates average fitted data curves from *n*=20 participants for (a) progesterone and (b) estrogen salivary concentrations after normalizing data to a standard 28 day cycle. Individual data points and fitted curves are shown in lighter grey. The triangle and circle markers indicate data points selected for direct comparisons between high and low progesterone comparisons shown in subsequent panels. Panel (c) compares ‘accuracy’ (ACC) rates and panel (d) compares ‘response times’ (RT) for high and low progesterone for left and right visual field (LVF/RVF) conditions. Each plot shows the median as the central line within the box, the box edges represent the 25^th^ and 75^th^ percentiles and the whiskers extend to most extreme data points not considered outliers. Outliers are plotted individually.
