# Supplementary material for: The Importance of the Derivative in Sex-Hormone Cycles: A Reason Why Behavioural Measures in Sex-Hormone Studies Are So Mercurial
Source: PLoS One. 2014 Nov 26;9(11):e111891. doi: 10.1371/journal.pone.0111891 (PMC4245079; doi:10.1371/journal.pone.0111891)
Supplement: File S4 — Visual field advantage over the menstrual cycle. (DOCX) [file pone.0111891.s004.docx]

Fluctuations in accuracy rates across the two visual fields were of particular interest as lateralization effects had been observed in previous studies.
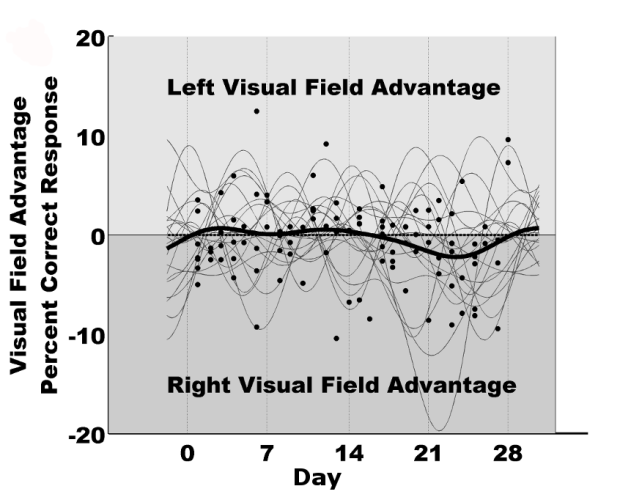


**Figure:** Visual field advantage over the menstrual cycle. The fitted average curve of the RVF-ACC subtracted from the average curve of the LVF-ACC data. It can be noted that on average there was little effect of visual field (main dark line) other than a small bias for RVF stimuli during the progesterone down sweep ~day 23. There was however a considerable range in difference between left and right VF performance across the cycle (mean range = 12.2% , min range = 5.5%, max range = 21.0%; within individuals lighter lines and data points shown). Light grey lines indicate an individual’s fitted RVF-ACC curve subtracted from the same individuals LVF-ACC fitted curve. Positive values indicate greater accuracy for LVF stimuli. The thick line indicates the group mean.
